# Patients’ Acceptance of Smartphone Health Technology for Chronic Disease Management: A Theoretical Model and Empirical Test

**DOI:** 10.2196/mhealth.7886

**Published:** 2017-12-06

**Authors:** Kaili Dou, Ping Yu, Ning Deng, Fang Liu, YingPing Guan, Zhenye Li, Yumeng Ji, Ningkai Du, Xudong Lu, Huilong Duan

**Affiliations:** ^1^ The Ministry of Education Key Laboratory of Biomedical Engineering College of Biomedical Engineering and Instrument Science Zhejiang University Hangzhou China; ^2^ School of Computing and Information Technology, Faculty of Engineering and Information Sciences University of Wollongong Wollongong Australia; ^3^ General Hospital of Ningxia Medical University Yinchuan China

**Keywords:** smartphone, mobile health, patients, hypertension, chronic disease, disease management

## Abstract

**Background:**

Chronic disease patients often face multiple challenges from difficult comorbidities. Smartphone health technology can be used to help them manage their conditions only if they accept and use the technology.

**Objective:**

The aim of this study was to develop and test a theoretical model to predict and explain the factors influencing patients’ acceptance of smartphone health technology for chronic disease management.

**Methods:**

Multiple theories and factors that may influence patients’ acceptance of smartphone health technology have been reviewed. A hybrid theoretical model was built based on the technology acceptance model, dual-factor model, health belief model, and the factors identified from interviews that might influence patients’ acceptance of smartphone health technology for chronic disease management. Data were collected from patient questionnaire surveys and computer log records about 157 hypertensive patients’ actual use of a smartphone health app. The partial least square method was used to test the theoretical model.

**Results:**

The model accounted for .412 of the variance in patients’ intention to adopt the smartphone health technology. Intention to use accounted for .111 of the variance in actual use and had a significant weak relationship with the latter. Perceived ease of use was affected by patients’ smartphone usage experience, relationship with doctor, and self-efficacy. Although without a significant effect on intention to use, perceived ease of use had a significant positive influence on perceived usefulness. Relationship with doctor and perceived health threat had significant positive effects on perceived usefulness, countering the negative influence of resistance to change. Perceived usefulness, perceived health threat, and resistance to change significantly predicted patients’ intentions to use the technology. Age and gender had no significant influence on patients’ acceptance of smartphone technology. The study also confirmed the positive relationship between intention to use and actual use of smartphone health apps for chronic disease management.

**Conclusions:**

This study developed a theoretical model to predict patients’ acceptance of smartphone health technology for chronic disease management. Although resistance to change is a significant barrier to technology acceptance, careful management of doctor-patient relationship, and raising patients’ awareness of the negative effect of chronic disease can negate the effect of resistance and encourage acceptance and use of smartphone health technology to support chronic disease management for patients in the community.

## Introduction

### Background

Due to its large impact on patients’ health status and health care expenditure, there is a growing interest worldwide in developing programs to support consumers to self-manage chronic diseases [1]. As the leading preventable risk factor for myocardial infarction, cerebral infarction, and heart failure, hypertension is an ongoing challenge to health care systems [2]. Patients’ self-management and self-care at home is essential for managing chronic diseases such as hypertension [3]. The ubiquitous smartphone technology provides a new opportunity for improving patients’ self-management of chronic diseases because it can enable frequent and flexible personal interaction with health care providers at the right time and right place [4]. A variety of smartphone health technologies have been reported worldwide to support different aspects of chronic disease management. There was evidence for smartphone technology to help patients improve blood pressure control [5] and medication adherence [6]. Some examples in hypertension management are patient self-recording of blood pressure [5,7], cardiovascular risk assessment [8,9], regular follow-up by doctors [10], health information recommendation [6], and automatic medication reminders [6]. Therefore, we developed a smartphone-based hypertension management app *Blood Pressure Assistant* and started a major project of introducing it to community-dwelling patients for hypertension management. The purpose of the program was to enable patients and their health care providers in a tertiary hospital to exchange information and collaborate in hypertension management.

Despite its potential benefits, mobile health (mHealth) technologies have encountered various challenges in patient acceptance [11]. According to a market report conducted in 27 countries in 2014, only 1.20% (1.6M/133M) of diabetic patients who had a smartphone were estimated to actually use a diabetes app on their smartphone to manage their disease [12]. For the successful introduction into the routine health care delivery system, it is essential to understand the factors impacting patients’ acceptance of the smartphone health technology.

Although there have been studies on consumer acceptance of health technology [13,14], the previous studies were focused on other technologies such as electronic medical records [15-18], telemonitoring technology [19], and the Web-based technology [20-22]. To the best of our knowledge, to date, little theoretically based technology acceptance study has been systematically conducted on smartphone technology. To fill this knowledge gap, this study aimed to develop and test a theoretical model to predict and explain patient acceptance of smartphone technology for chronic disease management. The theoretical model is tested in the context of hypertension management.

### Prior Research and Hypotheses

We conducted preliminary interviews with 10 patients who were frequent users of the smartphone health app Blood Pressure Assistant to understand why they used it. We identified 3 factors influencing their usage behavior: the need for hypertension control, compliance with their health care providers’ advice, and the reluctance to use it. This preliminary knowledge was taken into account in our conceptualization of the research model. The other constructs of the model were drawn from the relevant theories such as technology acceptance model (TAM) [23], TAM2 [24], dual-factor model [25], and health belief model (HBM) [26].

#### Technology Acceptance Model

Among its wide adoption in all fields of technology acceptance studies, TAM [23] has been used to predict consumer acceptance of health technology [13,20,27,28]. According to TAM, perceived usefulness and perceived ease of use are the 2 major cognitive determinants of information technology usage [13], such as consumer acceptance of smartphone health technology [23]. Perceived usefulness refers to the extent to which users believe that using a particular system would enhance their task performance. Perceived ease of use is the extent to which users believe that using a particular system would be easy [23]. Intention to use refers to the intention or the continual intention to use the technology. Combining TAM with the other models, Sun et al formulated a model to explain consumer acceptance of health technology [20]. Hung and Jen employed TAM to explore students’ intention to adopt mobile technology to manage personal health [27]. If patients believe that the alternative smartphone health technology is easy to use and will help with self-management of chronic disease, they will be more likely to adopt the technology. Moreover, if they feel the technology is easy to use, they would be more likely to perceive the technology as useful [29]. These expectations lead us to hypothesize the following:

H1: Perceived usefulness is positively associated with patients’ intention to use smartphone health technology.

H2: Perceived ease of use is positively associated with patients’ intention to use smartphone health technology.

H3: Perceived ease of use is positively associated with patients’ perceived usefulness of smartphone health technology.

Realizing the limitation of TAM in not considering the social factors that very much likely would influence a person’s perceptions about the technology, Venkatesh extended TAM to TAM2, which includes social influence (SI) as a key determinant of perceived usefulness and use intention [30]. SI is the degree to which the users perceive that the people who they trust and resort to believe they should use the technology. People are likely to incorporate trusted referents’ beliefs into their own belief structure [31], and therefore, we propose the following:

H4: Social influence is positively associated with perceived usefulness of smartphone health technology.

Moreover, users’ prior technology usage experience can shape their belief in the new technology [32]. Positive experience may help them to feel more confident and perceive that they have the capabilities and resources to repeat that same performance [28,32]. Therefore, we propose the following hypotheses:

H5a: The prior mobile app usage experience is positively associated with patients’ perceived usefulness of smartphone health technology.

H5b: The prior mobile app usage experience is positively associated with patients’ perceived ease of use of smartphone health technology.

#### Dual-Factor Model

Cenfetelli developed a dual-factor model of information technology usage to compensate the limitation of TAM being solely focused on users’ positive (enabling) perceptions but ignoring the negative (inhibiting) ones [25]. The core argument is that potential users’ information technology usage considerations are based on a simultaneous examination of both enabling and inhibiting factors. Cenfetelli contends that inhibitors not only influence information technology usage directly but also indirectly via enablers as the mediators [25].

Resistance to change (RTC) refers to people’s attempt to maintain their previous behaviors and habits in the face of change required. A study into physicians’ resistance toward health information technology finds that resistance to change is the inhibitor that has significant, direct influence on both behavioral intention and perceived usefulness [15]. Another study on older people’s acceptance of preventive mobile health services in China only finds the significant influence of resistance to change on perceived usefulness, not behavioral intention [33]. As patients were used to their familiar chronic disease management model, “social inertia” would likely cause them to have negative cognitive and emotional responses to the new smartphone health technology; thus, they may give relatively low evaluation on the technology’s usefulness. Thus, we propose the following hypotheses:

H6a: Resistance to change is negatively associated with intention to use smartphone health technology.

H6b: Resistance to change is negatively associated with perceived usefulness of smartphone health technology.

#### Health Belief Model

In essence, adoption of smartphone health technology is a patient’s behavior to promote, protect, or maintain their own health [20]. Therefore, it can also be explained by HBM [26], which suggests that people’s beliefs about health problems, perceived benefits of action and barriers to action, and self-efficacy explain engagement or lack of it in health promotion behavior [26]. In this study, perceived health threat refers to patients’ awareness and care of hypertensive condition, and its potential consequences. According to the previous literature, perceived health threat has both direct and indirect influences on consumer’s intention to use health information technology through perceived usefulness [13,15]. We thus propose the following hypotheses:

H7a: Perceived health threat is positively associated with patients’ intention to use smartphone health technology.

H7b: Perceived health threat is positively associated with perceived usefulness of smartphone health technology.

The perceived benefits of action in HBM are embodied in perceived usefulness in our new model. Barriers to action are modeled as resistance to change. Self-efficacy is the extent of patients’ beliefs in their ability to complete various tasks and reach the goal of controlling hypertensive condition. In the social cognitive theory (SCT), self-efficacy refers to users’ confidence in their ability to use a technology, and has been modeled as a determinant of perceived ease of use [30]. The definition of self-efficacy in the HBM includes that in SCT in this study context. In view of the logic, we propose the following hypothesis:

H8: Self-efficacy is positively associated with patients’ perceived ease of use of smartphone health technology.

#### Relationship With Doctor

The positive effects of doctor-patient interaction for chronic disease management have long been established [34]. Patient-doctor relationship is an important factor affecting patients’ e-health system adoption intention [35]. As found from the preliminary interview, health care providers play a vital role in guiding patients’ practices of chronic disease management. Patients who trusted the doctor’s expertise were more likely to communicate with the doctor whenever blood pressure arose. Therefore, these patients were more likely to appreciate the technology’s usefulness and ease of use for communication and were less likely to have negative resistance to technology. Therefore, we hypothesize the following:

H9a: Relationship with doctor is positively associated with perceived usefulness of the smartphone health technology.

H9b: Relationship with doctor is positively associated with perceived ease of use of the smartphone health technology.

H9c: Relationship with doctor is negatively associated with patients’ resistance to change.

#### Demographic Factors

A systematic review of studies on patient acceptance of consumer-centered health information technologies reveals that the most studied demographic variables on technology acceptance include sex, gender, and education [11]. Gender and age were found to be moderators between perceived usefulness and behavioral intention to use telemedicine service [36]. Age and education level appeared to have influenced consumers’ choice of use or nonuse of the e-appointment service [37]. 

**Figure 1:**
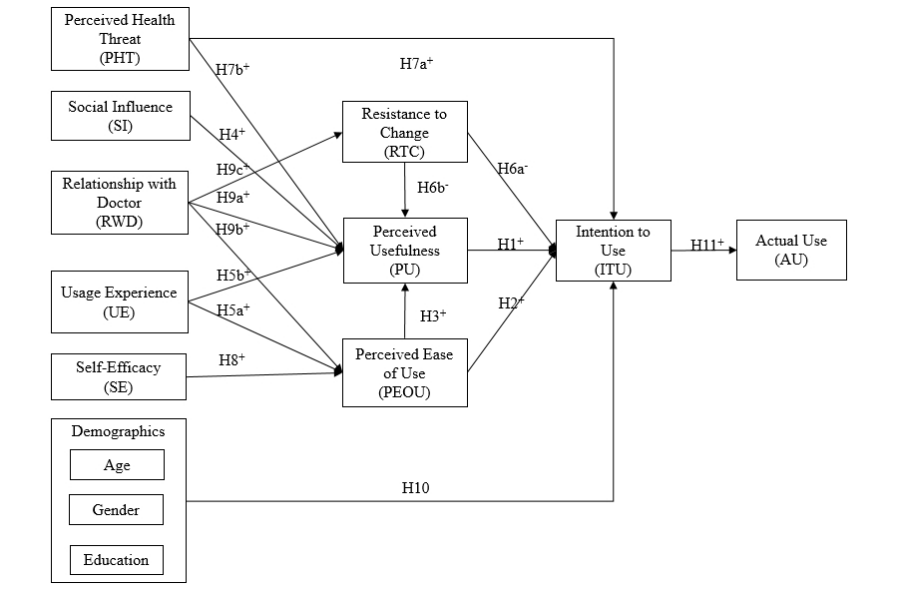
The hypothesized theoretical research model.

Thus, we tested the moderating effects of these 3 variables on intention to use and propose the following hypothesis:

H10a: Age is significantly associated with intention to use.

H10b: Gender is significantly associated with intention to use.

H10c: Education is significantly associated with intention to use.

#### Actual Use

In this study, we define actual use as the ratio of a patient’s actual use of the app to that prescribed in their management plan for a certain period of time. The predictive power of TAM is undermined if actual use is not included in the model [38] because intention is neither behavior nor is it necessarily translated into behavior. Therefore, there is a need to test whether intention is indeed translated into use. We propose the following hypothesis:

H11: Patients’ intention to use the smartphone health technology is positively associated with actual use.

### The Proposed Theoretical Research Model

On the basis of the above reasoning, we propose that 4 social factors—resistance to change, social influence, perceived health threat, and relationship with doctor—and a technical factor (ie, perceived ease of use), and a personal factor (ie, smartphone usage experience) affect patients’ perceived usefulness of smartphone health technology. Three factors, relationship with doctor, usage experience, and self-efficacy, affect patients’ perceived ease of use of the technology. Perceived usefulness, perceived ease of use, perceived health threat, and resistance to change affect patient’s intention to use. Three demographic variables, gender, age, and education, mediate the effect of the above variables on intention to use. Ultimately, intention to use affects patients’ actual use of smartphone health technology (Figure 1).

## Methods

### The Hypertension Management Program Enabled by the Smartphone Health Technology

**Figure 2:**
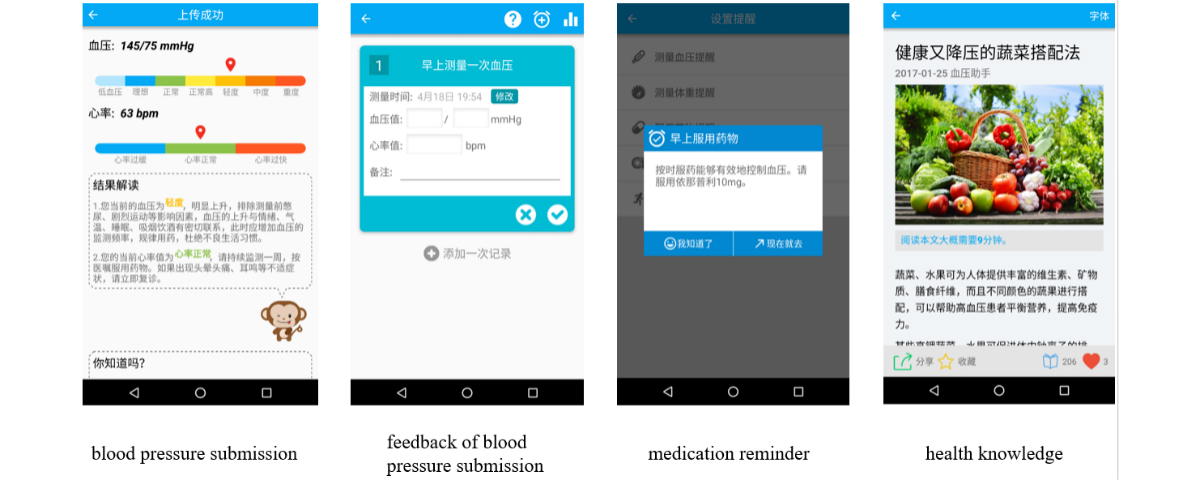
Screenshots of the smartphone-based Blood Pressure Assistant application.

The smartphone health app Blood Pressure Assistant was developed by the Biomedical Informatics Laboratory in Zhejiang University, People's Republic of China. It was designed to enable communication and collaboration between the outpatients and their health care providers in hypertension management. It included a smartphone app for the patients to use, named Blood Pressure Assistant (Figure 2), and a Web-based physician portal for their health care providers to communicate with these patients. The iPhone operating system (iOS) version of the app is downloadable from the Apple Store. Both the iOS and the Android version can be downloaded from a certain website. The physician portal also can be accessed at another certain website (if you want to use this application, please contact the author).

The functions of the smartphone app for patients included a reminder for blood pressure measurement, medication, and exercise; the form to enter and submit blood pressure measurement records; and receiving physician feedback and access to the health information published through the app.

The functions of the physician portal included continuous monitoring of patient health data, data visualization and reminding of abnormal situations, assessing patient health conditions based on the collected data, classification of patients according to their health conditions, and management of regular follow-up.

### Study Site

To improve population health, a chronic disease management program had been piloted to develop a model for chronic disease prevention and control in Ningxia Province in China. As the primary health care system was still in the emerging stage of development in the province, the program was run by the 2000-bed General Hospital of Ningxia Medical University, the only tertiary hospital in the province. The initial focus of the program was hypertension management. Therefore, the study population was the hypertensive outpatients in the Department of Cardiovascular Medicine in the hospital.

The health care providers who participated in the hypertension management program included a cardiovascular medicine specialist and a certified health manager. The program started once an outpatient was recruited and the hypertension management plan was developed for the person. A patient was requested to submit the blood pressure data via smartphone regularly according to the care plan. The system would assess whether the data were normal. An alarm would be flagged to the health care providers once any abnormal data were recorded. The health manager would then phone the patient to discuss the person’s abnormal health condition, reevaluate, and adjust the self-management plan. The patients could also read the information about chronic disease management published on the smartphone app. The system went live in November 2015.

### Recruiting the Study Participants

The health care providers recruited the outpatients into the program. Patients who met the following inclusion criteria were recruited: (1) aged 18 years or over; (2) no other serious complications except hypertension; (3) had a smartphone and sufficient network connectivity at home; (4) able to read and write in Chinese; and (5) resided in Yinchuan city so as to be contactable. After being recruited by the specialist, the health manager provided face-to-face training to the patients. The content of the training included knowledge about hypertension self-management, and the method to download “Blood Pressure Assistant” and use it, either from Apple Store if the person used an iPhone or from the specific website if the person used an Android phone [39]. Further information about the app can be acquired from the corresponding author. The training session usually lasted for 1 hour.

### Conducting Questionnaire Survey

Questionnaire survey was conducted between June and September 2016, 1 month after a patient entered the program. It was conducted either through the telephone survey or the electronic questionnaire survey.

We started conducting telephone interviews to collect questionnaires. A researcher made a phone call to an eligible patient. After informing the person about the survey and seeking the respondent’s oral consent, the researcher read and sought the person’s answer to each question, and then entered the answer into the electronic questionnaire survey form. After collecting 23 responses, we found this method to be resource-intensive and not efficient. Therefore, we piloted the method of using the mobile phone app to conduct the electronic questionnaire survey. In this method, a patient could fill in the electronic questionnaire survey form that automatically displayed on the smartphone health app interface 1 month after the person was recruited into the program. The information presented included the survey purpose, its voluntary nature, and insurance about anonymity of results in any related research publications. A patient could tick the check box to give consent. Implicit consent was assumed if a patient sent the completed questionnaire survey form back without ticking the check box to express consent.

After collecting 23 copies of electronic questionnaire responses, a *t* test was conducted to identify significant differences in results between the two data collection methods. As no difference was found, the rest of the data were collected via the electronic questionnaire survey.

The researchers extracted the questionnaire responses from the database for data analysis. In addition, data about each respondent’s actual use of the app were obtained from the system log in the database. The person’s number of interactions with the smartphone health app was tracked over a 7-day period, including 3 days before and 3 days after the day of response to the questionnaire. At the time of the survey, the system log only tracked the number of times a patient submitted the blood pressure (systolic and diastolic pressure) measurement data. Therefore, the patient’s actual use of the smartphone health app was calculated as the ratio of the number of times of submitting blood pressure measurement to the recommended number of times of submission in 7 days in the management plan.

### Measurements

A total of 24 questionnaire items were used to measure the 11 constructs in the theoretical model. These items were drawn from the previous validated instruments. A 5-point Likert scale was used for measurement, ranging from 1, strongly disagree, to 5, strongly agree (Table 1). The measurement items were translated into Chinese by 1 researcher, then discussed and validated by 5 researchers. One researcher back-translated the Chinese version into English.

The questionnaire was piloted on 5 patients to test the content validity. All of the measurement items except 1, “I am able to use Blood Pressure Assistant without much effort,” were easy for the patients to understand. We modified the “effort” into “time and energy” to improve readability. The patients’ demographic information was also collected, including age, gender, and education.

### Data Analysis

The research model was tested by the partial least squares (PLS) path modeling, a well-established statistical method to model the relationship between variables in social sciences, econometrics, marketing, and strategic management [27,41]. PLS modeling is a second-generation multivariate technique used to analyze causal models involving multiple constructs with multiple observed items. 

**Table 1:** The constructs, measurement items, and source references of the measurement items.

Construct	Item code	Measurement items	Source reference^a^
Demographics	—	Age, gender, and education	—
Perceived usefulness (PU)	PU1	Logging or sending blood pressure values would make me cope with hypertension better	[19]
	PU2	Knowing that a doctor checks my blood pressure data gives me confidence in hypertension management	
	PU3	Overall, Blood Pressure Assistant is useful	
Perceived ease of use (PEOU)	PEOU1	Learning how to use the mobile app would be easy for me	[39]
	PEOU2	I would find Blood Pressure Assistant easy to use	
	PEOU3	Blood Pressure Assistant is not cumbersome to use	
Social influence (SI)	SI1	People who are important to me think that I should use Blood Pressure Assistant	[40]
	SI2	People who are important to me use Blood Pressure Assistant	
Usage experience (UE)	UE1	I use smartphone to search health information on the Web	[35]
	UE2	I use mobile apps to help with managing health issues	
Resistance to change (RTC)	RTC1	I do not want the mobile app to change the way I deal with hypertension	[15]
	RTC2	I do not want the mobile health app to change the way I interact with other people	
Perceived health threat (PHT)	PHT1	I am aware of my hypertension condition	Drafted by authors
	PHT2	I am very concerned about hypertension	
	PHT3	I would take effort to manage hypertension	
Self-efficacy (SE)	SE1	I am able to use Blood Pressure Assistant without much time and energy	[20]
	SE2	I get the best value from using Blood Pressure Assistant	
Relationship with doctor (RWD)	RWD1	Doctors are my most trusted source of health information	[35]
	RWD2	When I have a health concern, my first step is to contact a doctor	
Intention to use (ITU)	ITU1	Given the opportunity, I would like to use Blood Pressure Assistant	[39]
	ITU2	I would consider to continuously use Blood Pressure Assistant	
Actual use (AU)	AU	Ratio of the actual number of measurements to the physician’s recommended number of measurements in care plan	—

^a^The symbol — denotes that the item has no source reference.

It is most suitable for models with relatively small samples in comparison with the covariance-based structural equation modeling technique [42]. This suits the case of our study. The data analysis was conducted in 2 stages. In stage 1, the reliability and validity of the constructs were evaluated. In stage 2, the structural model was tested.

### Ethics Approval

The Ethics Committee of the study hospital claimed that since this study did not involve patient data, there was no need for an ethics audit.

## Results

### The Demographic Results

There were 279 patients who used the system for more than 1 month. One hundred and fifty-two (54.5% (152/279) of them completed the questionnaire survey: 30 through telephone and 127 via electronic questionnaire. Giving 18 scale items to be tested, according to the minimum sample requirement of 5:1 subject-to-parameter, 90 questionnaire responses were sufficient for the PLS modeling [43]. Therefore, the sample size of 152 patients is deemed adequate. The general characteristics of the participating patients are shown in Table 2.

### Measurement Validation

Composite reliability (CR) and indicator reliability were used to assess the reliability of reflective constructs. All the constructs had adequate CR (ranged from 0.822 to 0.935) and indicator reliability (ranged from 0.710 to 0.976), both exceeding the recommended value of 0.70 [33]. Table 3 shows the descriptive statistics of the variables and the reliability coefficients.

**Figure 3:**
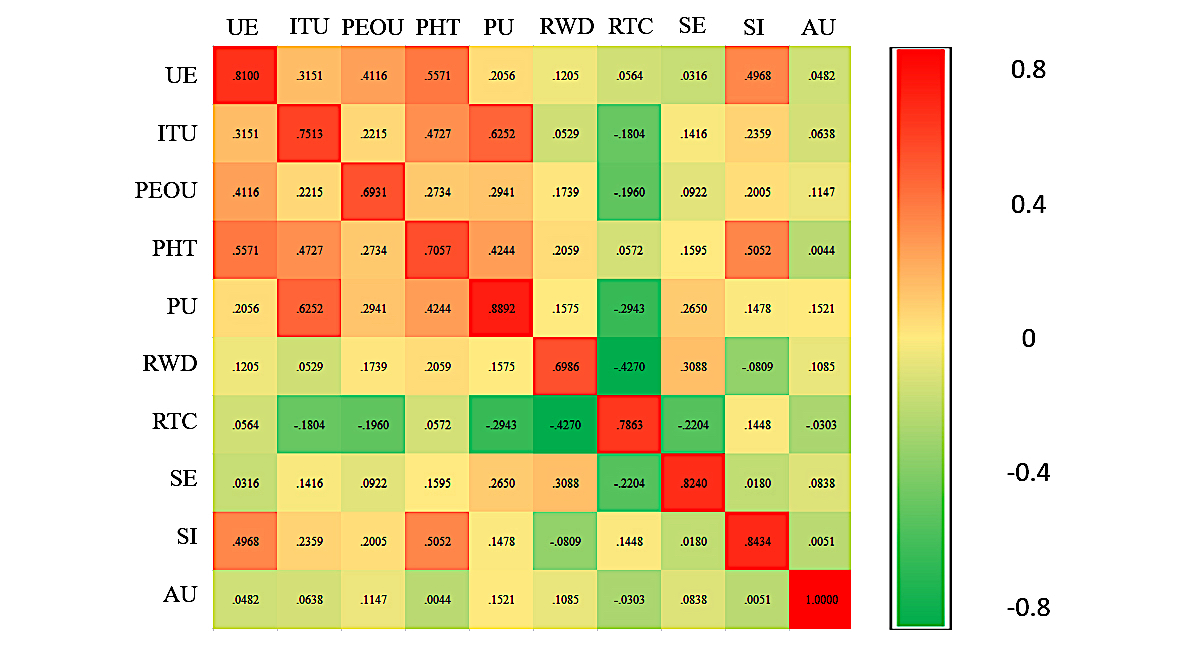
A heat map showing correlations and discriminant validity. The diagonal elements denote the square root of average variance extracted, and all other elements are correlations between the constructs.

The average variance extracted (AVE) of the construct was higher than the threshold of 0.50, confirming the convergent validity. AVE of each latent construct was higher than the construct’s highest squared correlation with any other latent construct (Figure 3), indicating the Fornell-Larcker criterion was met and confirming the discriminant validity.

### Model Validation

**Figure 4:**
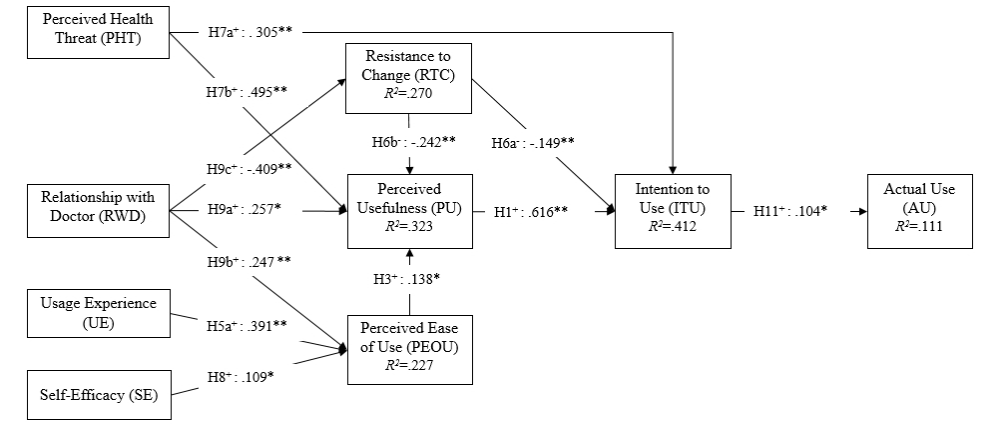
The validated theoretical model. *P<.05, **P<.01.

The model was assessed by checking the significance of path coefficients (β) among the independent variables and the latent variables. The demographic variable education was excluded from modeling because of large number of missing values. The variables age and gender were found to not have any significant influence on intention to use. The results of the PLS modeling are shown in Figure 4. In general, the model explained 0.412 of the total variance of intention to use and 0.111 of the variance in actual use.

**Table 2:** Demographics of the participating patients.

Characteristics		n (%)
**Gender**		
	Male	106 (69.7)
	Female	46 (30.3)
**Age in years**		
	<30	5 (3.2)
	<40	15 (9.9)
	40-49	55 (36.2)
	50-59	57 (37.5)
	>60	20 (13.2)
**Education**		
	<Middle school	9 (5.9)
	Middle school	12 (7.9)
	Vocational and technical education	18 (11.8)
	High school	25 (16.4)
	Three-year college	34 (22.4)
	University	38 (25)
	Missing information	16 (10.6)
**Users of different types of mobile phone**		
	iPhone operating system users	20 (13.2)
	Android users	132 (86.8)

**Table 3:** Descriptive statistics of the variables and the reliability coefficients.

Construct	Items	Mean (SD)	Standardized loading	Composite reliability
Usage Experience (UE)	UE1	3.23 (1.56)	.945	.8948
	UE2	3.07 (1.77)	.835	
Relationship with doctor (RWD)	RWD1	4.59 (0.74)	.870	.8223
	RWD2	4.40 (0.76)	.801	
Perceived health threat (PHT)	PHT1	4.13 (0.87)	.762	.8775
	PHT2	3.29 (2.05)	.863	
	PHT3	4.35 (0.63)	.890	
Perceived ease of use (PEOU)	PEOU1	4.58 (0.79)	.908	.8702
	PEOU2	4.26 (1.13)	.866	
	PEOU3	4.49 (0.84)	.710	
Perceived usefulness (PU)	PU1	4.17 (1.19)	.942	.9413
	PU2	4.68 (0.55)	.944	
Resistance to change (RTC)	RTC1	1.87 (1.25)	.921	.8802
	RTC2	1.66 (1.09)	.852	
Self-efficacy (SE)	SE1	4.33 (1.02)	.889	.9035
	SE2	4.47 (0.62)	.926	
Social influence (SI)	SI1	2.42 (1.98)	.944	.9150
	SI2	1.76 (1.93)	.891	
Intention to use (ITU)	ITU1	4.53 (0.94)	.955	.9350
	ITU2	4.64 (0.56)	.976	
Actual use (AU)	AU1	0.84 (0.13)	1	1

With a loading factor of 0.616, perceived usefulness had a major, significant positive effect on intention to use (β=.616, *t*_151_=6.203), supporting H1. Perceived ease of use had no significant effect on intention to use but a significant influence on perceived usefulness (β=.138, *t*_151_=2.335); thus, H2 was not supported but H3 was. Resistance to change (H6b), perceived health threat (H7b), and relationship with doctor (H9a) had significant associations with perceived usefulness (β=−.242, *t*_151_=3.058; β=.495, *t*_151_=3.180; and β=.257, *t*_151_=2.357, respectively) but not for social influence (H4) and prior technology usage experience (H5b). Therefore, H6b, H7b, and H9a were supported but H4 and H5b were rejected. Relationship with doctor (H9b), prior mobile app usage experience (H5a), and self-efficacy (H8) showed strong positive effects on perceived ease of use (β=.247, *t*_151_=2.685; β=.391, *t*_151_=4.092; and β=.110, *t*_151_=1.987, respectively). Both perceived health threat (H7a) and resistance to change (H6a) had a significant effect on intention to use (β=.305, *t*_151_=2.718 and β=−.149, *t*_151_=2.781, respectively). Moreover, relationship with doctor was found to have a significant negative effect on resistance to change, thus supporting H9c (β=−.409, *t*_151_=3.628). The demographic factors were found to have no significant impact on intention to use, thus the H10 was not supported. Intention to use (H11) was found to have a significant, weak influence on actual usage (β=.104, *t*=1.981).

## Discussion

### Principal Findings

This study proposed a hybrid smartphone health TAM for chronic disease management. The model was developed based on an extensive review of the related models and theories, including TAM [23], TAM2 [24], dual-factor model [25], and HBM [26]. The statistical measurements (composite reliability, indicator reliability, and AVE) supported the model’s reliability and validity. As the study participants used a specific smartphone health technology Blood Pressure Assistant for communication with their health care providers to manage hypertension, their relationship with doctors was considered an important antecedent factor in the model. The validity of the model was warranted by the survey participants being actual patients who had 1 month or more experience in using the smartphone health app for hypertension management. Several key findings emerge from this study.

First, as hypothesized, the antecedent variables—including resistance to change, perceived health threat, relationship with doctor, usage experience, and self-efficacy—influenced the patients’ acceptance of the smartphone health technology for hypertension management, along with the traditional TAM constructs, perceived usefulness and perceived ease of use. As these factors are considered by HBM to influence patients’ engagement in health promotion behavior, therefore, our finding supports the applicability of HBM in explaining patients’ behavior in using smartphone health technology for chronic disease management.

Moreover, 0.323 of variance in the perceived usefulness was explained by 3 variables: perceived health threat, relationship with doctor, and resistance to change. First, there were cascading effects starting from perceived health threat, to perceived usefulness, and to behavioral intention. The effect of perceived health threat was also found by Kim et al [13], who suggested that awareness and concern about deteriorating health conditions can motivate people to take action toward disease self-management.

Second, a major contribution of this study is to validate the significant influence of 2 antecedent factors, relationship with doctor and perceived health threat of hypertension, on the 3 intermittent factors for intention to use: the significant positive influences on both perceived usefulness and perceived ease of use, and strong negative influence on resistance to change. This demonstrated the vital role the health care providers play in any intervention that requires patients to self-manage their chronic diseases. In this study, the patients held highly positive evaluation of their relationships with doctors. This was suggested by their agreement with the statements that “doctors are my most trusted source of health information,” which scored 4.59 out of 5, and “When I have a health problem, my first step is to contact a doctor,” which scored 4.40 out of 5. These positive feelings were likely to be derived from the full attention and excellent service they received from the health care providers. They received 1-hour personal training from the health manager on hypertension management about how to download and use the smartphone app. If any abnormal blood pressure recording was reported, the health care providers would call the patients to discuss and adjust the hypertension management plan. These positive interactions built up rapport and patients’ trust with the health care providers. The trust could enhance the patient’s interest in using the smartphone health technology to communicate with the health care providers. Therefore, patients valued the usefulness of the technology.

This high evaluation of the relationship with doctor also led to the highly positive evaluation of the intermediate factors, perceived ease of use, and intention to use. It also strongly impeded resistance to change, with values for both items “I don’t want the mobile app to change the way I deal with hypertension” and “I don’t want the mobile health app to change the way I interact with other people” laid at the very low level between strongly disagree and disagree.

Third, the study confirmed that resistance to change indeed had a biasing effect on patients’ perception of usefulness of the smartphone technology. Its negative direct effect on behavioral intention was in accordance with that found in the middle-aged Chinese people’s acceptance of mobile health services, but not in the older people aged 60 years and above [31]. Its indirect negative effect on behavioral intention through the mediation of perceived usefulness was consistent with the previous studies on consumer acceptance of eHealth technology [22]. This reflected a natural tendency for some patients to prefer to continue with the traditional way of hypertension management than switching to use the new smartphone technology.

In accordance with the previous literature [13,28,44], both self-efficacy and smartphone technology usage experience had significant positive influence on the perceived ease of use. A sense of self-efficacy appeared to increase the likelihood for the patients to evaluate the technology to be easy to use. Their previous experience with any smartphone technology also provided them with the confidence with the new health app.

Perceived usefulness and perceived ease of use are 2 significant predictors of intention to use in the previous literature [27,28,31,45]. Consistent with the previous findings about consumer acceptance of eHealth technology [13,20,27,31], perceived usefulness was also found to be the most important predictor on intention to use. Therefore, to encourage hypertensive patients to adopt smartphone health technology to manage hypertension, we need to convince them that the technology is useful for them.

However, different from the previous studies’ findings [13,20,27,31,40], perceived ease of use had no significant effect on patients’ intention to use smartphone health technology. This difference in finding might be related to the different experiences the study participants had with the targeted technology. For example, Sun et al [20] conducted the study immediately after the participants were introduced to the technology, when they were still in the process of learning and familiarizing themselves with the technology. In this learning process, ease of use might be an important consideration for acceptance. Our survey was conducted after the patients had 1 month and more experience with the technology. They might be already familiar with it; thus, ease of use was no longer important for them. This was demonstrated by the very positive responses to the following statements: “Learning mobile app would be easy for me,” which scored 4.58 out of 5; “I would find Blood Pressure Assistant easy to use,” which scored 4.26 out of 5; and “Blood Pressure Assistant is not cumbersome to use,” which scored 4.49 out of 5. Another possible reason might be the increased prevalence of smartphone, leading to the general public’s increased familiarity with the smartphone apps. Thus, the technology was no longer seen as difficult to learn and use.

Contrary to the suggestion from the previous literature [13,20], social influence had no significant relationship with perceived usefulness. One possible explanation was that as a recently emerging consumer health technology, the smartphone health app was yet to be known by the general public. Therefore, the people who were the close referents to the survey participants had not yet had knowledge or formed their view about the technology. Thus, they did not have much influence on the respondents’ mobile health app usage behavior. This observation was supported by the low average score of the responses to the statement “People who are important to me think that I should use Blood Pressure Assistant,” which was only between “disagree and neutral” (2.42). The average score of the responses to the item stating “People who are important to me think that I should use Blood Pressure Assistant” was between “strongly disagree” to “disagree” (1.76). Another possibility was that after having hands-on usage experience with the technology for 1 month or more, the respondents’ attitudes toward it were no longer influenced by the significant others around. They made judgment based on their own experiences.

Finally, 1 step further from the previous consumer health technology acceptance studies [13,20,27,31,40,44], this study linked the input variables that measured individual beliefs to the actual use of a smartphone health app. It validated that intention to use had significant, weak relationship with actual use, explaining 0.111 of the variance in patients’ actual use of the smartphone health technology. This was contrary to Lim et al’s finding of a gap between intention and actual use [28]. This provides support to the validity of our tested theoretical model in explaining patient’s acceptance of smartphone health app.

### Limitations

This study is, understandably, limited by its empirical scope of the study population, their social, economic, and geographic location; the smartphone health app to be used; and the type of chronic disease they suffered from. The results may vary from place to place [40] or from the app in use to the other.

The measurement of constructs can be further developed. For example, relationship with doctor may include multiple aspects, in addition to the 2 items measured in this study. Previous studies found that privacy concern is an important factor influencing patients’ acceptance of information technology [46,47]. With portability and small size, use of smartphone app may indeed generate new security and privacy issues such as leakage and tampering of data transmitted over the wireless network [48], or stolen or lost device. Therefore, there is a need for all the sensor readings to be anonymized before analyzing them to guarantee the privacy of the participants [49]. As our research model did not include privacy concern as a construct, a future research direction is to integrate the privacy concern into the theoretical model.

Another limitation was the means by which the study participants were recruited. As only the patients who already used the smartphone health app Blood Pressure Assistant were recruited into the study, they were the innovative group of patient population who were likely to have a higher level of social economic status to afford to have smartphone than others; therefore, although the finding was valid for this population group, it may not be generalizable to the entire patient population even in our study site.

Only a moderate level of variation in use (0) was explained. The study captured actual use from only 1 dimension, patients’ submission of blood pressure recording. It did not capture use of other functions, such as accessing health educational information. Future research can identify other constructs influencing patients’ smartphone health technology use. The internal validity of the study was also confined by nonrespondents. Therefore, the future study can fine-tune the measurement of use. It also needs to evaluate the relationship between actual use and outcomes.

### Conclusions

The study developed a theoretical model about patients’ acceptance of smartphone health technology for chronic disease management. It found that patients’ perceived usefulness of smartphone health technology was positively influenced by their perceived health threat, relationship with doctor, and perceived ease of use, but negatively influenced by resistance to change. Good patient-doctor relationships can alleviate patient resistance to change. Usage experience and self-efficacy positively influenced patients’ perceived ease of use. Intention to use was influenced by the enablers of perceived usefulness and perceived health threat, and the inhibitor of resistance to change. Intention to use had a significant, weak relationship with actual use.

### Implications for Practice

Although the rapid growth of smartphone technology has opened new opportunities for chronic disease management, the opportunity can only be captured by the patients who accept and use the technology. The findings suggest that 3 antecedent factors, relationship with doctor, perceived health threat, and resistance to change, are important for patients’ acceptance and use of smartphone health technology. Therefore, for the successful introduction of smartphone health technology innovation for chronic disease management, efforts need to be focused on improving patient-doctor relationship and providing continuous patient education to raise awareness of the disease’s threat to health. These strategies will be effective in overcoming potential resistance to change and encouraging acceptance and use of the new technology.

## Abbreviations

AU: actual use
AVE: average variance extracted
CR: composite reliability
HBM: health belief model
ITU: intention to use
mHealth: mobile health
PEOU: perceived ease of use
PHT: perceived health threat
PLS: partial least square
PU: perceived usefulness
RTC: resistance to change
RWD: relationship with doctor
SCT: social cognitive theory
SE: self-efficacy
SI: social influence
TAM: technology acceptance model
UE: usage experience

## References

[1] Franek J (2013). Self-management support interventions for persons with chronic disease: an evidence-based analysis. Ont Health Technol Assess Ser.

[2] James PA, Oparil S, Carter BL, Cushman WC, Dennison-Himmelfarb C, Handler J, Lackland DT, LeFevre ML, MacKenzie TD, Ogedegbe O, Smith Jr SJ, Svetkey LP, Taler SJ, Townsend RR, Wright Jr JT, Narva AS, Ortiz E (2014). 2014 evidence-based guideline for the management of high blood pressure in adults: report from the panel members appointed to the Eighth Joint National Committee (JNC 8). J Am Med Assoc.

[3] Clark NM (2003). Management of chronic disease by patients. Annu Rev Public Health.

[4] Free C, Phillips G, Felix L, Galli L, Patel V, Edwards P (2010). The effectiveness of M-health technologies for improving health and health services: a systematic review protocol. BMC Res Notes.

[5] Or C, Tao D (2016). A 3-month randomized controlled pilot trial of a patient-centered, computer-based self-monitoring system for the care of type 2 diabetes mellitus and hypertension. J Med Syst.

[6] Bengtsson U, Kjellgren K, Hallberg I, Lindwall M, Taft C (2016). Improved blood pressure control using an interactive mobile phone support system. J Clin Hypertens (Greenwich).

[7] Hervás R, Fontecha J, Ausín D, Castanedo F, Bravo J, López-de-Ipiña D (2013). Mobile monitoring and reasoning methods to prevent cardiovascular diseases. Sensors (Basel).

[8] Liu Z, Chen S, Zhang G, Lin A (2015). Mobile phone-based lifestyle intervention for reducing overall cardiovascular disease risk in Guangzhou, China: a pilot study. Int J Environ Res Public Health.

[9] Najafi SS, Shaabani M, Momennassab M, Aghasadeghi K (2016). The nurse-led telephone follow-up on medication and dietary adherence among patients after myocardial infarction: a randomized controlled clinical trial. Int J Community Based Nurs Midwifery.

[10] Kang H, Park HA (2016). A mobile app for hypertension management based on clinical practice guidelines: development and deployment. JMIR Mhealth Uhealth.

[11] Or CK, Karsh BT (2009). A systematic review of patient acceptance of consumer health information technology. J Am Med Inform Assoc.

[12] Research 2 Guidance.

[13] Kim J, Park HA (2012). Development of a health information technology acceptance model using consumers' health behavior intention. J Med Internet Res.

[14] Pai FY, Huang KI (2011). Applying the technology acceptance model to the introduction of healthcare information systems. Technol Forecast Soc Change.

[15] Bhattacherjee A, Hikmet N (2007). Physicians' resistance toward healthcare information technology: a theoretical model and empirical test. Eur J Inf Syst.

[16] Hennington A, Janz BD (2007). Information systems and healthcare XVI: physician adoption of electronic medical records: applying the UTAUT model in a healthcare context. Commun Assoc Inf Syst.

[17] Tang PC, Ash JS, Bates DW, Overhage JM, Sands DZ (2006). Personal health records: definitions, benefits, and strategies for overcoming barriers to adoption. J Am Med Inform Assoc.

[18] Winkelman WJ, Leonard KJ, Rossos PG (2005). Patient-perceived usefulness of online electronic medical records: employing grounded theory in the development of information and communication technologies for use by patients living with chronic illness. J Am Med Inform Assoc.

[19] Buysse HE, Coorevits P, Van Maele G, Hutse A, Kaufman J, Ruige J, De Moor GJ (2010). Introducing telemonitoring for diabetic patients: development of a telemonitoring 'Health Effect and Readiness' questionnaire. Int J Med Inform.

[20] Sun Y, Wang N, Guo X, Peng JZ (2013). Understanding the acceptance of mobile health services: a comparison and integration of alternative models. J Electron Commer Res.

[21] Kim D, Chang H (2007). Key functional characteristics in designing and operating health information websites for user satisfaction: an application of the extended technology acceptance model. Int J Med Inform.

[22] Or CK, Karsh BT, Severtson DJ, Burke LJ, Brown RL, Brennan PF (2011). Factors affecting home care patients' acceptance of a web-based interactive self-management technology. J Am Med Inform Assoc.

[23] Davis FD (1989). Perceived usefulness, perceived ease of use, and user acceptance of information technology. MIS Quarterly.

[24] Venkatesh V, Davis FD (2000). A theoretical extension of the technology acceptance model: four longitudinal field studies. Manage Sci.

[25] Cenfetelli RT (2004). Inhibitors and enablers as dual factor concepts in technology usage. J Assoc Inf Syst.

[26] Janz NK, Becker MH (1984). The health belief model: a decade later. Health Educ Behav.

[27] Hung MC, Jen WY (2012). The adoption of mobile health management services: an empirical study. J Med Syst.

[28] Lim S, Xue L, Yen CC, Chang L, Chan HC, Tai BC, Duh HB, Choolani M (2011). A study on Singaporean women's acceptance of using mobile phones to seek health information. Int J Med Inform.

[29] Lazard AJ, Watkins I, Mackert MS, Xie B, Stephens KK, Shalev H (2016). Design simplicity influences patient portal use: the role of aesthetic evaluations for technology acceptance. J Am Med Inform Assoc.

[30] Venkatesh V (2000). Determinants of perceived ease of use: integrating control, intrinsic motivation, and emotion into the technology acceptance model. Inf Syst Res.

[31] Deng Z, Mo X, Liu S (2014). Comparison of the middle-aged and older users' adoption of mobile health services in China. Int J Med Inform.

[32] Taylor S, Todd PA (1995). Assessing IT usage: the role of prior experience. MIS Quarterly.

[33] Guo X, Sun Y, Wang N, Peng Z, Yan Z (2013). The dark side of elderly acceptance of preventive mobile health services in China. Electron Markets.

[34] Kaplan SH, Greenfield S, Ware Jr JE (1989). Assessing the effects of physician-patient interactions on the outcomes of chronic disease. Med Care.

[35] Koopman RJ, Petroski GF, Canfield SM, Stuppy JA, Mehr DR (2014). Development of the PRE-HIT instrument: patient readiness to engage in health information technology. BMC Fam Pract.

[36] Rho MJ, Kim HS, Chung K, Choi IY (2014). Factors influencing the acceptance of telemedicine for diabetes management. Cluster Comput.

[37] Zhang X, Yu P, Yan J, Ton AMSI (2015). Using diffusion of innovation theory to understand the factors impacting patient acceptance and use of consumer e-health innovations: a case study in a primary care clinic. BMC Health Serv Res.

[38] Turner M, Kitchenham B, Brereton P, Charters S, Budgen D (2010). Does the technology acceptance model predict actual use? A systematic literature review. Inf Softw Technol.

[39] Yu P, Li H, Gagnon MP (2009). Health IT acceptance factors in long-term care facilities: a cross-sectional survey. Int J Med Inform.

[40] Dwivedi YK, Shareef MA, Simintiras AC, Lal B, Weerakkody V (2016). A generalised adoption model for services: a cross-country comparison of mobile health (m-health). Gov Inf Q.

[41] Arteaga F, Gallarza MG, Gil I, Vinzi VE, Chin WW, Henseler J, Wang H (2010). A new multiblock PLS based method to estimate causal models: application to the post-consumption behavior in tourism. Handbook of Partial Least Squares.

[42] Hair JF, Ringle CM, Sarstedt M (2011). PLS-SEM: indeed a silver bullet. J Mark Theory Pract.

[43] Bentler PM, Chou CP (1987). Practical issues in structural modeling. Sociol Methods Res.

[44] Wilson EV, Lankton NK (2004). Modeling patients' acceptance of provider-delivered e-health. J Am Med Inform Assoc.

[45] Deng Z (2013). Understanding public users' adoption of mobile health service. Int J Mob Commun.

[46] Avancha S, Baxi A, Kotz D (2012). Privacy in mobile technology for personal healthcare. ACM Comput Surv.

[47] Katz JE, Rice RE (2009). Public views of mobile medical devices and services: a US national survey of consumer sentiments towards RFID healthcare technology. Int J Med Inform.

[48] Meingast M, Roosta T, Sastry S (2006). Security and privacy issues with health care information technology. Conf Proc IEEE Eng Med Biol Soc.

[49] Mayora O, Frost M, Arnrich B, Gravenhorst F, Grünerbl A, Muaremi A, Osmani V, Puiatti A, Reichwaldt N, Scharnweber C, Tröster G (2016). Pdfs.semanticscholar.

